# EPHX2 Expression and Its Association with Prognosis, Metabolic Regulation, and Metastasis-Related Pathways in Lung Adenocarcinoma

**DOI:** 10.3390/genes17080961

**Published:** 2026-08-16

**Authors:** Şebnem Yıldırımcan Kadıçeşme

**Affiliations:** Department of Medical Biology, Faculty of Medicine, Kafkas University, Kars 36100, Turkey; sebnem.yildirimcan@kafkas.edu.tr

**Keywords:** *EPHX2*, lung adenocarcinoma, prognostic biomarker, arachidonic acid metabolism, angiogenesis, bioinformatics

## Abstract

**Background/Objectives**: Epoxide hydrolase 2 (*EPHX2*), which encodes soluble epoxide hydrolase (sEH), is involved in arachidonic acid metabolism and has been associated with inflammation, lipid metabolism, and tumor biology. However, its prognostic significance and biological associations in lung adenocarcinoma (LUAD) remain unclear. This study aimed to investigate the expression profile, prognostic value, and molecular pathways of *EPHX2* in LUAD using bioinformatics analyses. **Methods**: EPHX2 expression was evaluated using TNMplot, GEPIA2, and GEO datasets, while protein expression was assessed using the Human Protein Atlas and CPTAC/UALCAN platforms. Prognostic analyses were performed using Kaplan–Meier Plotter, GEPIA2, and Human Protein Atlas datasets. Co-expression and gene set enrichment analyses were conducted using LinkedOmics, and functional enrichment analyses were performed using Gene Ontology (GO), Kyoto Encyclopedia of Genes and Genomes (KEGG), and Reactome databases. Correlation and protein–protein interaction (PPI) analyses were evaluated using GEPIA2 and STRING. **Results**: *EPHX2* expression was significantly reduced in LUAD tissues compared with normal lung tissues across datasets, and these findings were supported at the protein level. High *EPHX2* expression was associated with better overall survival and retained independent prognostic significance in multivariate Cox analysis. Functional enrichment analyses demonstrated associations with lipid metabolism, arachidonic acid metabolism, cytochrome P450-related pathways, and oxidative processes. Correlation analyses suggested potential associations between *EPHX2* and angiogenesis, extracellular matrix remodeling, and hypoxia-related pathways. **Conclusions**: Bioinformatics analyses suggest that *EPHX2* may participate in metabolic and tumor progression-related regulatory networks and may serve as a prognostic biomarker in LUAD.

## 1. Introduction

Lung cancer remains one of the leading causes of cancer-related deaths worldwide, with lung adenocarcinoma (LUAD) standing out as the most common histological subtype of non-small-cell lung cancer (NSCLC) [1]. Despite advances in diagnosis and treatment, the prognosis in LUAD patients remains heterogeneous, and there is an ongoing need for reliable biomarkers capable of predicting clinical outcomes [2]. Therefore, a better understanding of the molecular biology of LUAD is critical for identifying novel prognostic biomarkers and therapeutic targets.

In recent years, metabolic reprogramming has been recognized as a key determinant of tumor progression in cancer biology [3]. In particular, lipid metabolism and arachidonic acid derivatives play a significant role in tumor cell proliferation, inflammation, angiogenesis, and metastasis [4,5]. In this context, epoxyeicosatrienoic acids (EETs) have garnered attention as biological mediators that influence vascular tone, cell migration, and the tumor microenvironment [6,7].

Soluble epoxide hydrolase (sEH), encoded by the *EPHX2* gene, plays a central role in the regulation of these biological signals by converting EETs into DHETs, which are less active diols [6,7]. The effect of *EPHX2* on this metabolic balance suggests that it may indirectly influence tumor biology through lipid signaling. Indeed, it has been shown that changes in EET levels modulate cancer-related processes such as angiogenesis, inflammation, and cell invasion [8].

In addition to their roles in lipid metabolism, arachidonic acid metabolites and lipid signaling pathways are increasingly recognized as key regulators of the tumor microenvironment, including hypoxia adaptation, extracellular matrix remodeling, and angiogenic signaling [3,9]. Growing evidence suggests that metabolic reprogramming in the tumor microenvironment may significantly influence tumor progression, immune modulation, and treatment response in LUAD [10].

However, the role of *EPHX2* in cancer has not yet been fully elucidated, and both tumor-suppressive and tumor-promoting effects have been reported in various tumor types [8,11,12]. In particular, the clinical significance of *EPHX2* expression and its underlying molecular mechanisms in LUAD have been investigated in only a limited number of studies. This suggests that *EPHX2*’s role in tumor progression may be context-specific and that more comprehensive analyses are needed. The evaluation of gene expression in cancer should not be limited to the single-gene level but must also be considered within the framework of interactions between genes and their transcriptional regulators and associated signaling pathways. In particular, it is known that transcription factors associated with metabolic processes and the oxidative stress response, such as *PPARA*, *RXRA*, and *NFE2L2*, play a critical role in the regulation of genes linked to lipid metabolism [13,14]. In this context, elucidating *EPHX2*’s role in these regulatory networks could contribute to a more comprehensive understanding of the gene’s potential biological functions and its role in tumor progression. Given the emerging role of metabolic reprogramming in shaping the tumor microenvironment, metabolism-associated genes such as *EPHX2* may also contribute to tumor adaptation and progression in LUAD.

In this study, the expression profile, prognostic value, and potential molecular mechanisms of the *EPHX2* gene in LUAD were systematically investigated using multi-step bioinformatics analyses. Additionally, by evaluating *EPHX2*-associated genes, protein–protein interaction networks, and functional pathways, the potential associations between this gene and pathways related to angiogenesis, invasion, and tumor progression were explored. Overall, this integrative approach provides a more comprehensive mechanistic framework for understanding the potential biological role of EPHX2 in LUAD.

## 2. Materials and Methods

### 2.1. Study Design and Workflow

In this study, a multi-step bioinformatics analysis approach was applied to investigate the expression profile, prognostic value, and potential molecular mechanisms of the *EPHX2* gene in LUAD. The study design was planned to include gene expression, survival, co-expression, functional enrichment, correlation, and protein–protein interaction analyses.

First, *EPHX2* gene expression was compared between tumor and normal tissue samples using the TNMplot [15], GEPIA2 [16], and Gene Expression Omnibus (GEO; GSE10072) [17] databases. Protein expression was validated using data from the Human Protein Atlas [18] and CPTAC/UALCAN [19].

Subsequently, survival analyses were performed using the Kaplan–Meier Plotter, GEPIA2, and Human Protein Atlas platforms to assess the clinical significance of EPHX2. Co-expression analysis was conducted on the LinkedOmics platform to identify genes associated with *EPHX2*, and gene set enrichment analysis (GSEA) was applied to the resulting gene list [20].

Functional enrichment analyses were performed using the Gene Ontology (GO), Kyoto Encyclopedia of Genes and Genomes (KEGG), and Reactome databases. Based on the enrichment results, the relationships between *EPHX2* and the selected representative genes were evaluated using correlation analyses. Protein–protein interaction (PPI) networks were constructed using the STRING platform for genes showing significant correlations, and the associated KEGG pathways were evaluated [21].

Finally, by integrating the findings from correlation, enrichment, and protein–protein interaction analyses, a hypothesis-generating association model was constructed to illustrate potential upstream regulators and downstream pathways associated with EPHX2. A summary of the datasets, analyses, and analysis parameters used in this study is provided in Supplementary Table S1.

### 2.2. Expression Profiling of EPHX2 in LUAD

EPHX2 gene expression levels were analyzed using the TNMplot, GEPIA2, and Gene Expression Omnibus (GEO; GSE10072) databases to compare tumor and normal tissue samples [15,16,17].

In the TNMplot database, matched normal and tumor tissues from LUAD were compared in RNA-seq analysis, while normal, primary tumor, and metastatic lung tissues were evaluated in microarray analysis.

On the GEPIA2 platform, *EPHX2* expression was compared between LUAD and normal lung tissues using TCGA and GTEx data. A |log2FC| cutoff of 1 and a *p*-value threshold of 0.01 were applied.

The GSE10072 dataset was analyzed as part of the GEO database. Differential gene expression analysis was performed using the GEO2R tool to compare LUAD and normal lung tissue samples. Statistical analyses included log2 fold change (logFC) and adjusted *p*-values, and an adjusted *p* < 0.05 was considered statistically significant. The findings were evaluated at the protein level using Human Protein Atlas data [18]. Additionally, EPHX2 protein expression was compared between LUAD and normal lung tissues using CPTAC proteomic data via the UALCAN platform [19,22]. Default parameters of the relevant platforms were used for all analyses.

### 2.3. Survival Analysis

The prognostic value of the *EPHX2* gene was evaluated using the Kaplan–Meier Plotter platform [23]. The LUAD cohort was selected for the analyses, and 1161 LUAD patients were included. Patients were divided into high- and low-expression groups based on the median EPHX2 expression value. The JetSet best probe set was selected, and EPHX2 expression was assessed using the 209368_at probe in all analyses. Overall survival (OS) analysis was performed using the Kaplan–Meier method, and survival curves were plotted along with 95% confidence intervals (CI). Hazard ratios (HR) and log-rank *p*-values were automatically calculated by the platform. A *p*-value < 0.05 was considered statistically significant.

In addition to the OS analysis in the LUAD cohort, univariate survival analyses were performed according to stage, tumor grade, T, N, and M stages, sex, smoking status, surgical margin status, chemotherapy, and radiotherapy.

A multivariate Cox regression analysis was performed to evaluate the independent prognostic value of *EPHX2*. Stage, sex, and smoking status were included as covariates in the multivariate model.

To independently validate the Kaplan–Meier Plotter results, additional overall survival analyses were performed using the LUAD datasets available through the GEPIA2 and Human Protein Atlas (version 18) platforms [16,18]. In the GEPIA2 survival analysis, the LUAD cohort was analyzed using the Overall Survival method. Patients were classified into high- and low-expression groups based on the median *EPHX2* expression value (50%/50% cutoff).

### 2.4. Co-Expression and Pathway Enrichment Analyses

To investigate the biological role of the *EPHX2* gene, RNA-seq data from the TCGA-LUAD cohort were analyzed using the LinkedOmics platform [20]. The relationships between *EPHX2* expression and other genes were evaluated using the Pearson correlation coefficient. Using the gene rankings obtained from the correlation analysis, WebGestalt-based GSEA was performed on the LinkedOmics platform. The GO, KEGG, and Reactome databases were utilized to identify enriched biological processes and signaling pathways.

In the GSEA, genes were ranked by Pearson correlation coefficients, and no predefined cutoff was applied. Statistical significance was assessed using the false discovery rate (FDR) method.

### 2.5. Correlation Analyses of EPHX2-Associated Genes

Following an assessment of the clinical significance of *EPHX2* expression, correlation analyses were conducted to investigate potential associations with metastasis-related pathways. In this context, correlation analyses were performed using metastasis-related marker genes involved in angiogenesis (*VEGFA*, *FLT1*, and *ANGPT2*), invasion (*MMP2* and *MMP9*), and epithelial–mesenchymal transition (EMT; *VIM* and *CDH1*), which are well established in the literature.

Following the identification of significant associations with *VEGFA*, *FLT1*, *ANGPT2*, *MMP2*, and *MMP9*, results from co-expression and functional enrichment analyses (GO, KEGG, and Reactome) were examined to more comprehensively evaluate the biological processes associated with *EPHX2*. Pathway analyses and literature data were combined, and representative genes known to play critical roles in the relevant biological processes were identified.

Accordingly, to more comprehensively evaluate potential downstream mechanisms associated with EPHX2, genes involved in hypoxia and EET signaling (*HIF1A*, *PIK3CA*, *AKT1*, *MTOR*, *RAF1*, *MAPK1*, and *MAPK3*), genes associated with angiogenesis (*KDR* and *TEK*), genes associated with cell adhesion and migration (*PTK2* and *ITGB1*), and genes associated with extracellular matrix remodeling (*TIMP1* and *TIMP2*) were included in the correlation analyses.

In addition, genes associated with lipid metabolism and nuclear receptor signaling (*PPARA*, *PPARG*, *RXRA*, *RXRB*, and *HNF4A*), genes associated with the oxidative stress response (*NFE2L2*), genes associated with inflammatory signaling pathways (*STAT3* and *RELA*), genes associated with transcriptional regulation (*SP1* and *CEBPA*), and genes associated with the xenobiotic response (*AHR*) were identified as potential upstream genes and included in the analyses.

Correlation analyses were performed on LUAD tumor samples using the GEPIA2 platform [16]. Relationships between gene expressions were calculated using the Spearman correlation coefficient. TPM-based expression data were used in the analyses, and the results were evaluated on a log2-transformed scale. A *p*-value < 0.05 was considered statistically significant. Because the correlation analyses were performed using a predefined panel of biologically relevant candidate genes selected based on enrichment analyses and prior literature, no multiple-testing correction was applied.

### 2.6. Protein–Protein Interaction Network and Functional Enrichment Analyses

PPI network analysis was performed using the STRING database (v12.0) [21]. The genes included in the analysis were grouped into two main categories: candidate downstream effector genes associated with *EPHX2* and candidate upstream regulatory genes.

As part of the downstream analysis, genes showing significant correlations in correlation analyses and associated with angiogenesis, cell adhesion/migration, and extracellular matrix (ECM) remodeling—namely *VEGFA*, *FLT1*, *ANGPT2*, *MMP2*, *MMP9*, *TIMP1*, *TIMP2*, *ITGB1*, *TEK*, and *HIF1A*—were included in the analysis.

As part of the upstream analysis, *EPHX2* was evaluated alongside *CEBPA*, *PPARA*, *RXRA*, *RXRB*, *NFE2L2*, and *STAT3*—genes identified as significant in correlation analyses and associated with transcriptional regulation. The minimum interaction score was set at 0.700 (high confidence), and only experimental data, database evidence, and co-expression sources were used. Text mining and other predictive data sources were excluded from the analysis. Network expansion was not applied, and only the queried proteins were evaluated.

EPHX2-centered functional enrichment analysis was performed using the STRING platform, and KEGG pathway results were evaluated. By integrating the findings from correlation, PPI, and functional enrichment analyses, a hypothesis-generating association model was developed that summarizes the upstream regulatory and downstream signaling pathways associated with EPHX2 in LUAD.

### 2.7. Statistical Analysis

Statistical analyses were performed using the analytical tools integrated into the respective online platforms. Correlations were evaluated using the Spearman correlation coefficient. Survival analyses were performed using the Kaplan–Meier method and the log-rank test; the Cox regression model was applied for multivariate analyses. A *p*-value < 0.05 was considered statistically significant. All publicly available datasets and online analysis platforms used in this study were accessed between April 2026 and May 2026.

## 3. Results

### 3.1. Expression Analysis of EPHX2 in LUAD

**Figure 1:**
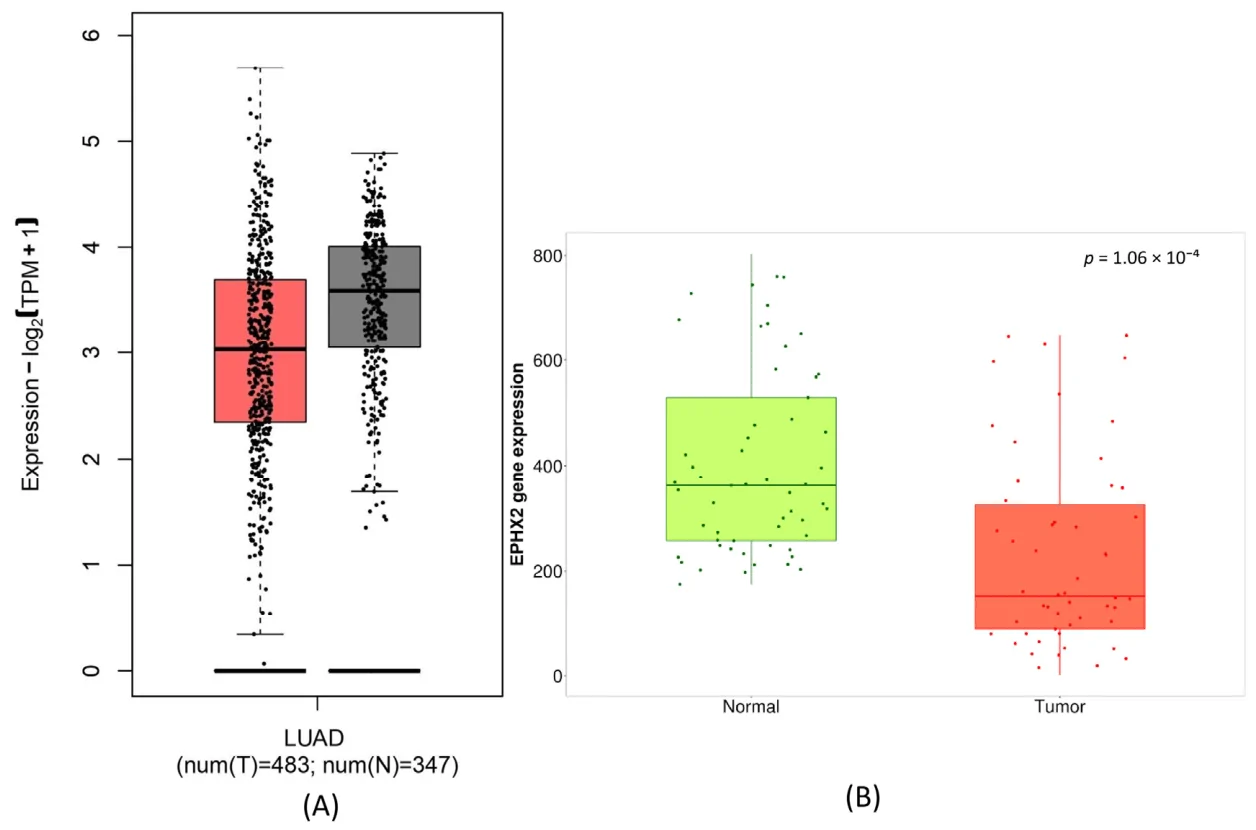
Evaluation of *EPHX2* mRNA expression in LUAD and normal lung tissues. (**A**) GEPIA2 analysis [16]. *EPHX2* expression was compared between LUAD tumor tissues (*n* = 483) and normal lung tissues (*n* = 347) using one-way ANOVA. (**B**) TNMplot RNA-seq analysis [15]. *EPHX2* expression was compared between matched normal and tumor tissues (*n* = 57 vs. 57) using the paired Wilcoxon test.

In the analysis conducted using the GEPIA2 platform, TCGA-LUAD tumor data (*n* = 483) were compared with normal lung tissues from TCGA and GTEx (*n* = 347), and it was found that *EPHX2* gene expression was significantly reduced in tumor tissues compared to normal tissues (*p* < 0.05) (Figure 1A).

In the TNMplot RNA-seq analysis, a comparison of matched normal and tumor tissues (*n* = 57 vs. *n* = 57) revealed that *EPHX2* expression was significantly lower in tumor tissues compared to normal tissues in LUAD samples (*p* < 0.001; FC = 0.67) (Figure 1B).

A total of 2264 lung tissue samples (1865 tumor, 391 normal, and 8 metastatic) were analyzed using TNMplot microarray analysis. *EPHX2* expression showed a decreasing trend from normal tissue to tumor and metastatic tissues (*p* < 0.001). In pairwise comparisons, *EPHX2* expression was significantly lower in tumor tissues than in normal tissues (Dunn’s test, *p* < 0.001; FC = 0.81). No significant difference was observed between metastatic and normal tissues (*p* = 0.065). Furthermore, no significant difference was detected between metastatic and primary tumor tissues (*p* = 0.385). However, the number of metastatic samples was limited (*n* = 8) (Supplementary Figure S1).

**Figure 2:**
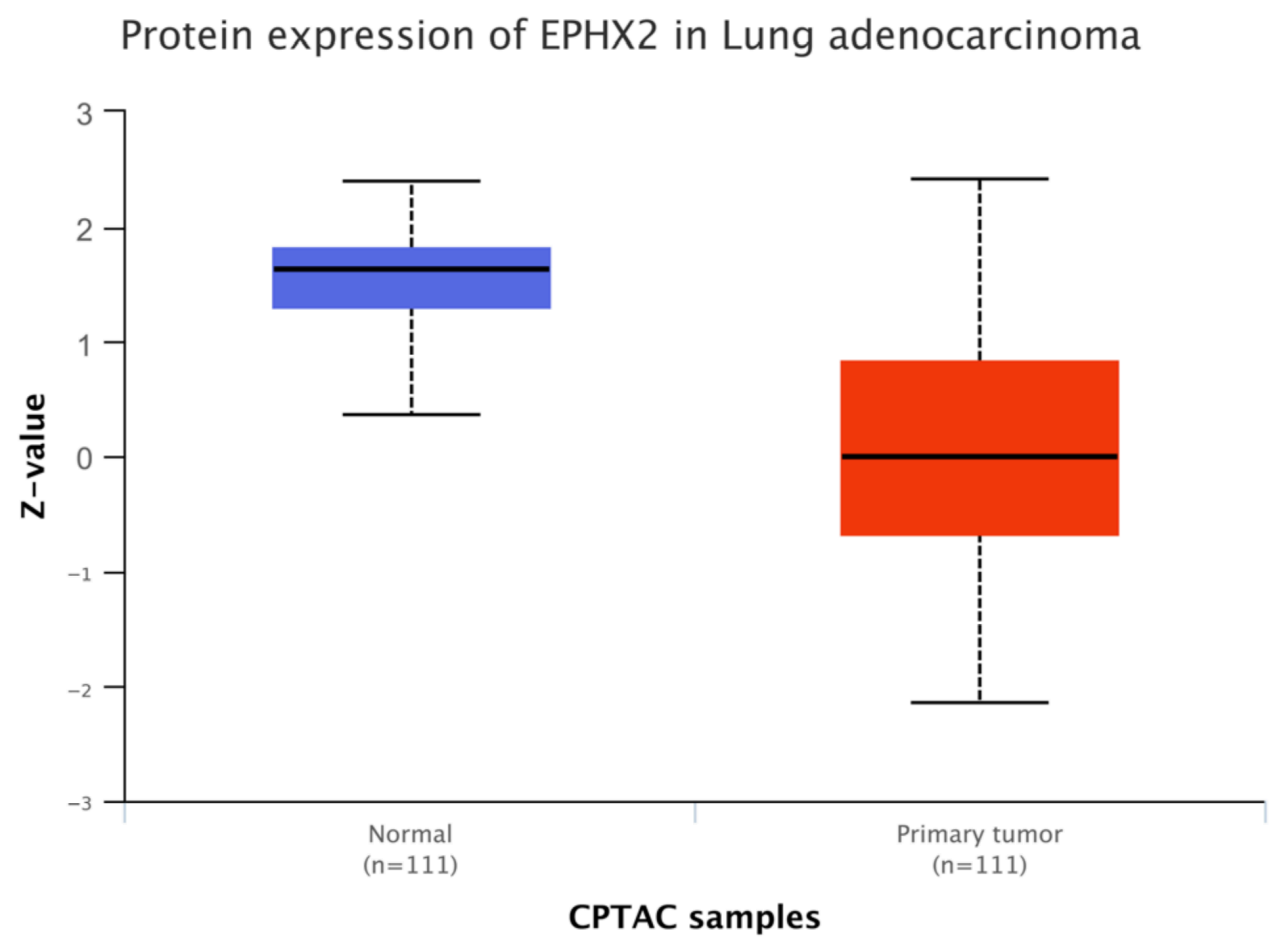
Comparison of EPHX2 protein expression in LUAD and normal lung tissue in a CPTAC-based UALCAN analysis [19,22]. Protein expression was compared between normal lung tissues (*n* = 111) and primary LUAD tissues (*n* = 111) using an unpaired two-sample *t*-test.

Unlike other transcriptomic datasets, a comparison of 47 normal and 58 tumor samples in the GEO GSE10072 dataset revealed that EPHX2 expression was significantly higher in tumor tissues (logFC = 0.33; adj. *p* < 0.001). This discrepancy may be related to platform differences, sample heterogeneity, or cohort-specific characteristics. Importantly, the overall consistency observed across multiple RNA-seq and proteomic datasets supports the robustness of the general expression trend despite inter-platform variability. At the protein level, Human Protein Atlas data showed that EPHX2 protein expression was significantly lower in LUAD tissues than in normal lung tissues (*p* < 0.001; Supplementary Figure S2) [18]. Similarly, UALCAN analysis based on CPTAC data (normal *n* = 111; tumor *n* = 111) also revealed that EPHX2 protein expression was significantly reduced in LUAD tissues (*p* < 0.001; Figure 2) [19,22].

### 3.2. Prognostic Significance of EPHX2 in LUAD

**Figure 3:**
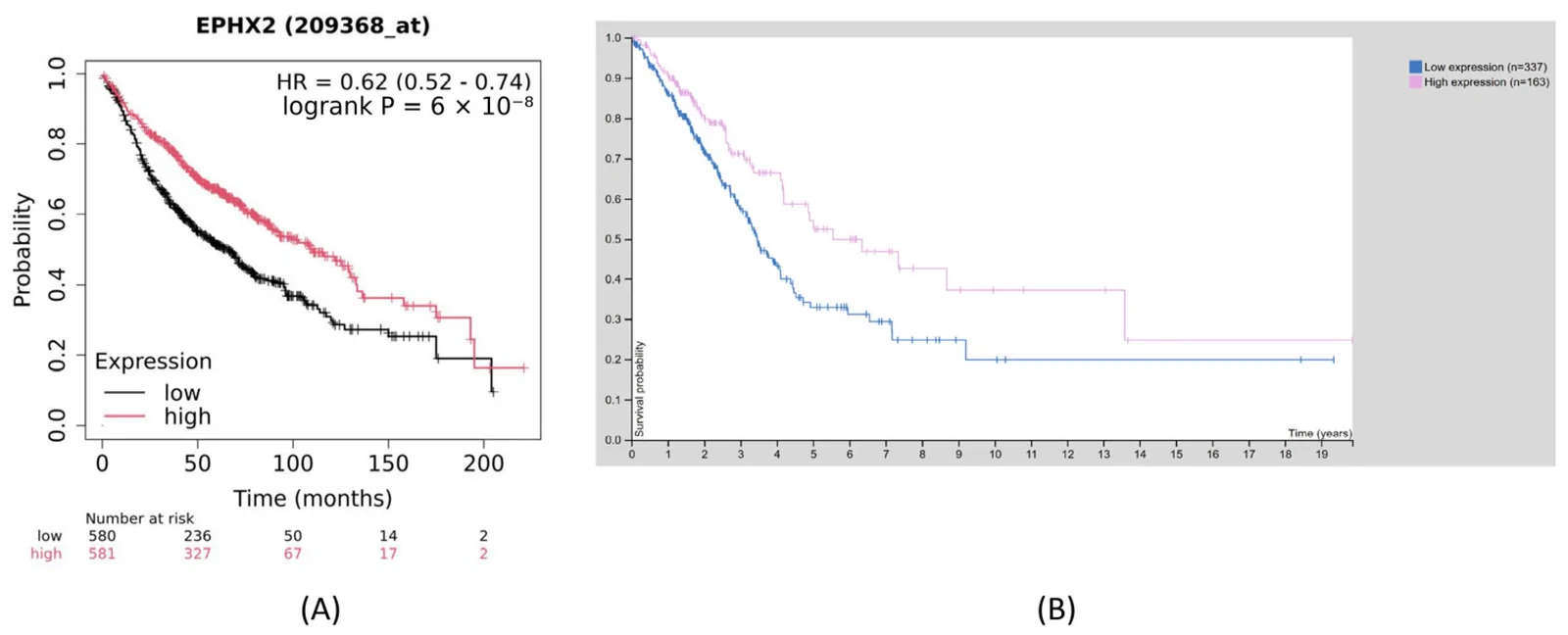
Overall survival analysis according to *EPHX2* expression in patients with LUAD. (**A**) Kaplan–Meier Plotter analysis [23]. Overall survival was evaluated in the LUAD cohort (*n* = 1161) using the log-rank test. (**B**) Human Protein Atlas analysis [18]. Overall survival was evaluated in the LUAD cohort (*n* = 500) using the log-rank test.

According to the Kaplan–Meier plot analysis, high *EPHX2* expression was significantly associated with better overall survival in patients with LUAD (*n* = 1161) (HR = 0.62, 95% CI: 0.52–0.74; *p* < 0.001) (Figure 3A). In the survival analysis conducted using the GEPIA2 platform, 478 patients with LUAD were evaluated, and no statistically significant association was found between *EPHX2* expression and overall survival (HR = 0.75; *p* = 0.057). In a survival analysis using Human Protein Atlas data, it was observed that high *EPHX2* expression was associated with better overall survival in LUAD patients (*n* = 500) (*p* = 0.0032) (Figure 3B) [18].

Subgroup analyses revealed that the prognostic impact of *EPHX2* expression in the LUAD cohort may vary depending on clinicopathological variables. In the analysis by stage, high *EPHX2* expression was significantly associated with better overall survival in Stage I patients (HR = 0.42, *p* < 0.001); however, this association was not maintained in advanced stages.

In the analysis by tumor grade, no significant association between *EPHX2* expression and survival was observed in any grade subgroup.

In the analysis based on tumor size, no statistically significant association was observed in the T1 (HR = 0.72, *p* = 0.083), T2 (HR = 0.79, *p* = 0.110), and T3 (HR = 0.78, *p* = 0.530) subgroups.

In analyses based on lymph node status, high *EPHX2* expression was significantly associated with better overall survival in N0 (HR = 0.72, *p* = 0.018) and N1 (HR = 0.60, *p* = 0.019) patients; however, no significant association was observed in N2 patients (HR = 0.96, *p* = 0.880). When evaluated by metastasis status, high *EPHX2* expression was found to be significantly associated with better overall survival in M0 patients (HR = 0.54, *p* = 0.002).

In clinical subgroup analyses, high EPHX2 expression was significantly associated with better overall survival in both female (HR = 0.58, *p* < 0.001) and male patients (HR = 0.67, *p* < 0.001). Furthermore, this association was significant in both never-smokers (HR = 0.51, *p* = 0.026) and smokers (HR = 0.69, *p* = 0.005). In patients with negative surgical margins, high *EPHX2* expression was also significantly associated with better overall survival (HR = 0.59, *p* < 0.001). Detailed results for the subgroups are presented in Table 1.

In the multivariate Cox regression analysis, tumor stage remained a significant prognostic factor (HR = 2.45, 95% CI: 1.77–3.39, *p* < 0.001). *EPHX2* expression was independently associated with overall survival after adjustment for stage, sex, and smoking status (HR = 0.64, 95% CI: 0.42–1.00, *p* = 0.048). Smoking status was also significantly associated with survival (HR = 0.58, 95% CI: 0.34–0.99, *p* = 0.047), whereas sex was not significantly associated with survival (HR = 0.89, 95% CI: 0.57–1.40, *p* = 0.621).

### 3.3. Co-Expression and Functional Enrichment Analyses

**Figure 4:**
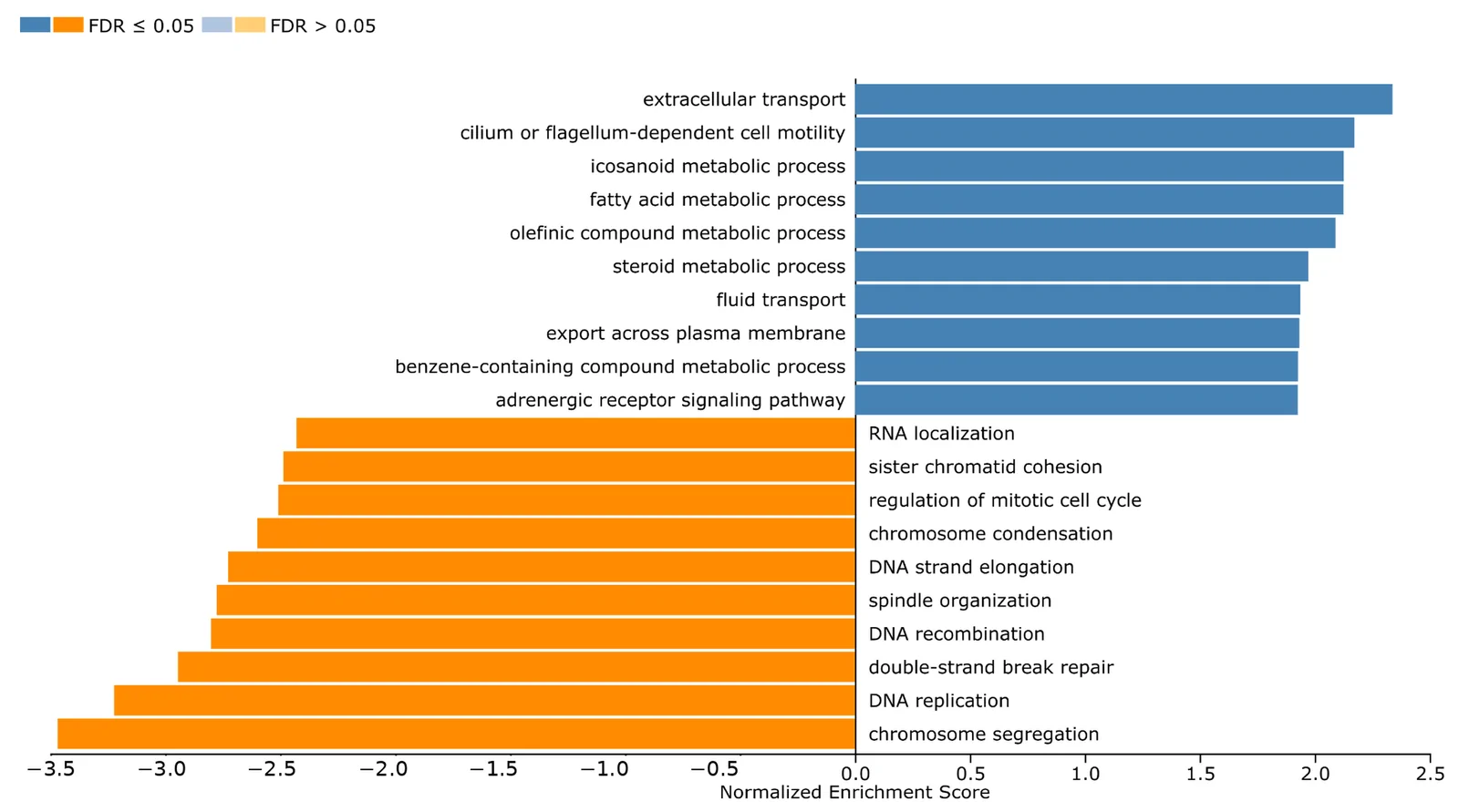
GO biological process enrichment analysis of genes correlated with EPHX2. The major biological processes showing positive and negative enrichment are presented according to normalized enrichment score (NES) values [20].

GSEA-based functional enrichment analyses using the GO, KEGG, and Reactome databases revealed that genes correlated with *EPHX2* were significantly enriched in pathways related to metabolism and detoxification. GO analysis revealed significant enrichment in lipid-related biological processes, particularly eicosanoid metabolic processes and fatty acid metabolism (FDR < 0.001). These findings suggest that EPHX2-associated genes are closely related to lipid metabolic processes (Figure 4). In addition, a significant enrichment was observed in processes related to extracellular transport (NES = 2.34, FDR < 0.001). Genes showing negative correlation were significantly enriched in processes related to the cell cycle, DNA replication, and DNA repair.

In the KEGG pathway analysis, genes correlated with EPHX2 were particularly enriched in metabolic pathways, and “drug metabolism” was the most significantly enriched pathway (NES = 2.48, FDR < 0.001). Additionally, pathways associated with arachidonic acid metabolism, cytochrome P450-mediated xenobiotic metabolism, and fatty acid metabolism were significantly enriched. Among the negatively enriched pathways, processes related to the cell cycle, DNA replication, and DNA repair were among the most significantly enriched pathways.

In the Reactome analysis, genes associated with *EPHX2* were notably enriched in oxidative metabolism and detoxification processes. The most significant enrichment was observed in the “biological oxidations” pathway (NES = 2.39, FDR < 0.001). In addition, pathways associated with Phase I metabolism, fatty acid metabolism, arachidonic acid metabolism, and peroxisomal lipid metabolism were significantly enriched. Among the negatively enriched pathways, processes related to the cell cycle, G1/S transition, and DNA replication/repair were predominant.

### 3.4. Correlation Patterns of EPHX2-Associated Genes

Correlation analyses revealed several weak-to-moderate associations between *EPHX2* expression and genes related to angiogenesis, extracellular matrix remodeling, hypoxia signaling, and transcriptional regulation. When genes associated with angiogenesis were examined, *EPHX2* expression showed a weak but significant negative correlation with *VEGFA* (r = −0.11, *p* = 0.012) and its receptor *FLT1* (r = −0.11, *p* = 0.019). In addition, a more pronounced negative correlation was observed with *ANGPT2* (r = −0.19, *p* < 0.001).

When genes associated with invasion and extracellular matrix remodeling were evaluated, *EPHX2* expression showed a weak negative correlation with *MMP2* (r = −0.095, *p* = 0.037) and a stronger negative correlation with *MMP9* (r = −0.20, *p* < 0.001).

In contrast, no statistically significant correlation was found between EPHX2 expression and the EMT-associated genes *VIM* and *CDH1* (*p* > 0.05). In correlation analyses evaluating potential downstream signaling pathways associated with *EPHX2*, varying degrees of association were identified among genes involved in hypoxia and EET-related signaling pathways. While a weak but significant negative correlation was observed between *HIF1A* and *EPHX2* (r = −0.16, *p* < 0.001), no significant relationship was detected with *PIK3CA*, *AKT1*, *MTOR*, and *MAPK1* (*p* > 0.05). In contrast, significant positive correlations were identified with *RAF1* (r = 0.23, *p* < 0.001) and *MAPK3* (r = 0.15, *p* < 0.001).

Among the genes associated with angiogenesis, a moderate positive correlation was observed with *TEK* (r = 0.24, *p* < 0.001), while no significant association was found with *KDR* (*p* > 0.05). Among genes involved in cell adhesion and migration, a moderate negative correlation was observed with *ITGB1* (r = −0.26, *p* < 0.001), and a weak positive correlation was observed with *PTK2* (r = 0.11, *p* = 0.019). Additionally, significant negative correlations were found between *EPHX2* and *TIMP1* (r = −0.22, *p* < 0.001) and *TIMP2* (r = −0.10, *p* = 0.025), which are associated with extracellular matrix regulation.

Correlation analyses also identified significant associations between *EPHX2* expression and several transcriptional regulators. The strongest positive correlations were observed with *CEBPA* (r = 0.35, *p* < 0.001), *STAT3* (r = 0.29, *p* < 0.001), *RXRB* (r = 0.27, *p* < 0.001), *RXRA* (r = 0.26, *p* < 0.001), *NFE2L2* (r = 0.25, *p* < 0.001), and *PPARA* (r = 0.24, *p* < 0.001).

In addition, weak but significant positive correlations were detected with *SP1* (r = 0.14, *p* = 0.003) and HNF4A (r = 0.14, *p* = 0.003). In contrast, no significant correlation was observed between *EPHX2* expression and *AHR*, *PPARG*, or *RELA* (*p* > 0.05). The complete list of statistically significant correlations with EPHX2 expression is presented in Supplementary Table S2.

### 3.5. Protein–Protein Interaction and Functional Enrichment Analyses

**Figure 5:**
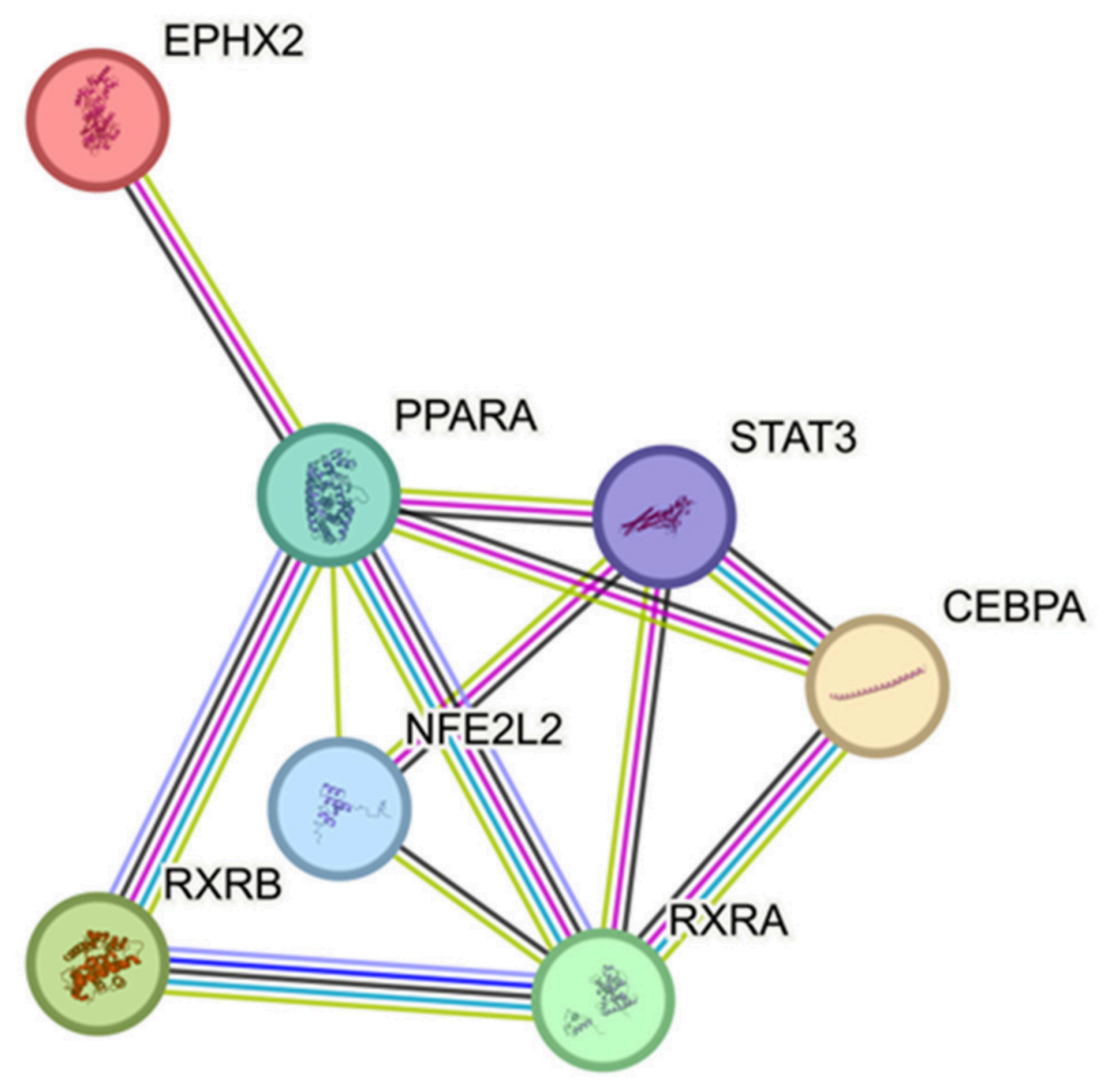
STRING-based protein–protein interaction network among upstream regulatory genes [21].

In the STRING-based protein–protein interaction analysis, strong connections were identified among PPARA, RXRA, and RXRB, particularly within the upstream regulatory network. Additionally, STAT3, NFE2L2, and CEBPA were also found to form dense interactions within the network. In contrast, EPHX2 did not exhibit strong direct interactions with other proteins and occupied a more isolated position within the network (Figure 5). These findings suggest that the upstream regulatory factors form an interconnected transcriptional regulatory network.

**Figure 6:**
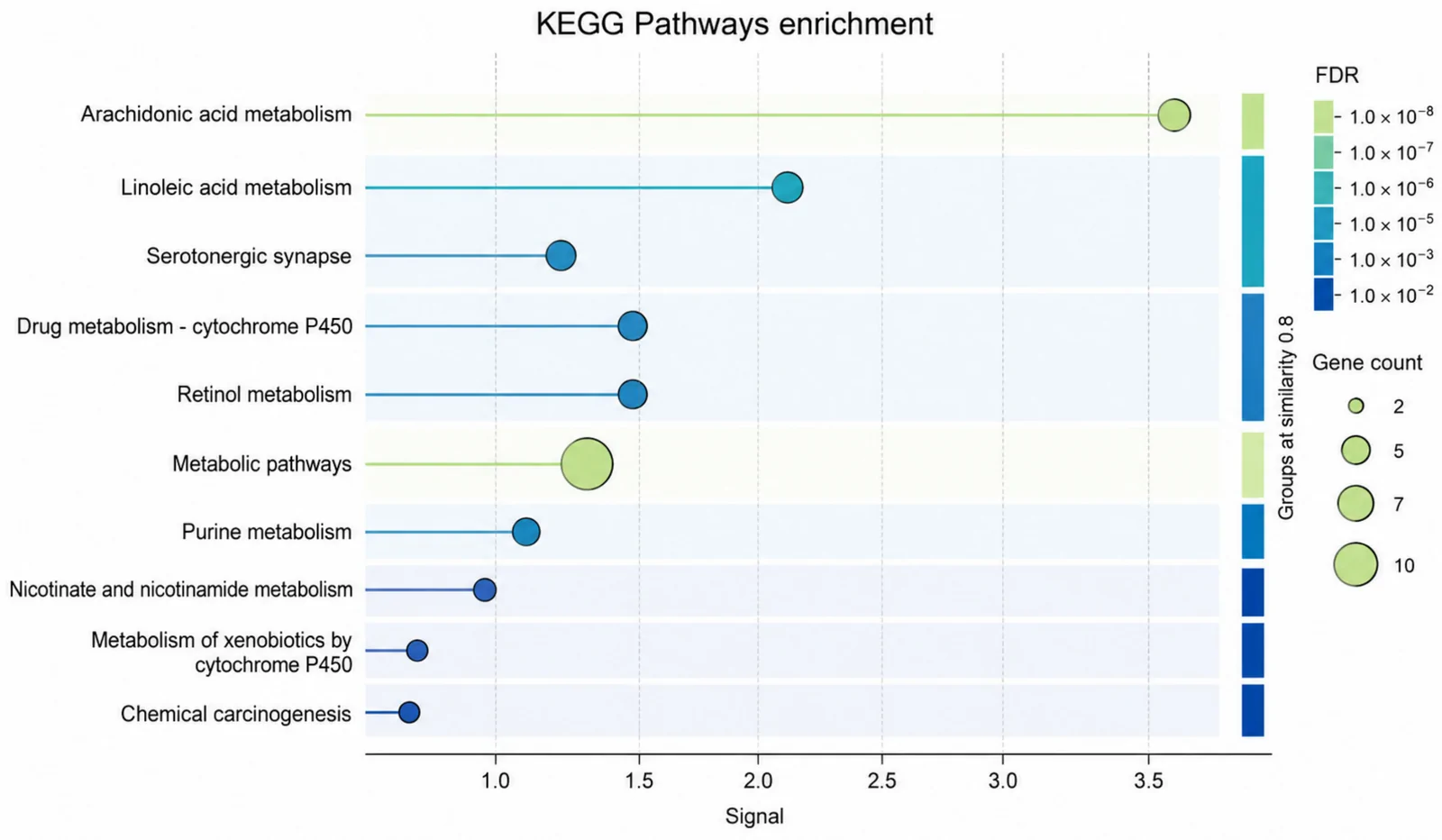
STRING-based KEGG pathway enrichment analysis of genes associated with EPHX2 [21].

In the STRING-based KEGG enrichment analysis, genes associated with EPHX2 were significantly enriched in arachidonic acid metabolism, linoleic acid metabolism, and metabolic pathways (Figure 6). The most significant enrichment was observed in the arachidonic acid metabolism pathway.

In the STRING-based protein–protein interaction analysis, distinct interaction networks were observed among VEGFA, FLT1, ANGPT2, MMP2, MMP9, TIMP1, TIMP2, ITGB1, TEK, and HIF1A, which were evaluated in the downstream analysis. In particular, strong connections were identified within the VEGFA–FLT1 axis as well as between the MMP2/MMP9 and TIMP1/TIMP2 pairs. Additionally, interactions between TEK and ANGPT2 were prominent in the angiogenesis-related network. In contrast, EPHX2 showed limited interactions with other proteins in the network and occupied a more isolated position (Supplementary Figure S3).

### 3.6. Proposed Hypothesis-Generating Model of EPHX2-Associated Signaling

**Figure 7:**
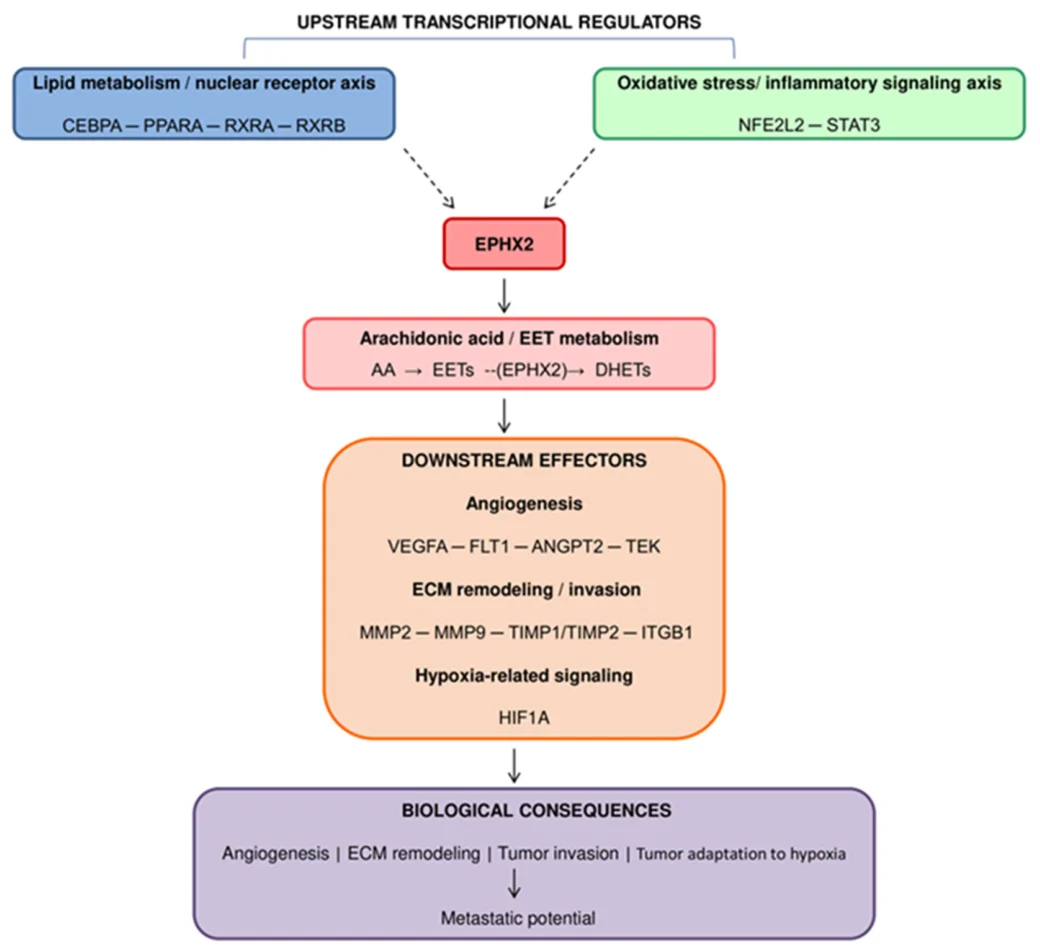
Proposed hypothesis-generating model of upstream regulatory and downstream signaling pathways associated with EPHX2 in LUAD. Note: The model was constructed based on correlation analyses, STRING-based protein–protein interaction network analyses, and functional enrichment findings. The relationships presented in the model are inferential and hypothesis-generating associations derived from bioinformatics analyses. Dashed arrows indicate potential regulatory associations, whereas solid lines represent potential biological relationships supported by the literature and biological network analyses.

By integrating the findings from correlation, STRING/PPI, and enrichment analyses, a hypothesis-generating model associated with EPHX2 was proposed (Figure 7). The proposed model suggests that EPHX2 may be regulated by upstream transcriptional regulators associated with lipid metabolism, oxidative stress, and inflammatory signaling pathways. Furthermore, through downstream effectors, EPHX2 expression may be associated with biological processes related to angiogenesis, extracellular matrix remodeling, hypoxia-related signaling, and metastatic progression. These findings are hypothesis-generating and require experimental validation.

## 4. Discussion

Lung adenocarcinoma is one of the malignancies for which there is a need to identify new prognostic biomarkers due to its high mortality rate and marked molecular heterogeneity [1,24]. In recent years, the role of tumor metabolism and lipid-mediated signaling pathways in cancer progression has attracted increasing attention [25,26]. In this study, the expression pattern, prognostic value, and potential molecular associations of the *EPHX2* gene in lung adenocarcinoma were evaluated using comprehensive bioinformatics analyses. To the best of our knowledge, studies that comprehensively evaluate the expression pattern, prognostic value, and associated molecular pathways of EPHX2 in LUAD at the transcriptomic and proteomic levels are quite limited.

The findings indicate that *EPHX2* expression is generally reduced in LUAD tumor tissues and that high *EPHX2* expression is associated with better overall survival. Furthermore, co-expression, correlation, and functional enrichment analyses suggest that *EPHX2* may be linked to biological pathways associated with lipid metabolism, arachidonic acid metabolism, oxidative processes, and tumor progression [26,27].

In this study, the finding that *EPHX2* expression was significantly lower in GEPIA2 and TNMplot analyses than in normal lung tissue suggests that EPHX2 may possess tumor-suppressing properties. It should be noted that the normal–tumor comparisons in GEPIA2 are based on integrated TCGA and GTEx datasets, and therefore potential batch effects between these data sources cannot be completely excluded. The fact that EPHX2 protein expression was significantly reduced in LUAD tissues in UALCAN analyses based on Human Protein Atlas and CPTAC proteomic data supports the findings obtained at the transcriptomic and protein level [18,22]. However, these protein expression data should be interpreted as supportive, descriptive evidence because HPA immunohistochemistry and CPTAC/UALCAN analyses cannot distinguish tumor cells from other cell types within the tissue and, therefore, cannot establish tumor-cell-specific downregulation of EPHX2. Although the literature reports that EPHX2 exhibits variable expression profiles in various cancer types, it has been suggested that the suppression of genes associated with lipid metabolism, particularly in solid tumors, may be linked to tumor progression [3,28]. The finding that low EPHX2 expression is associated with a poor prognosis in hepatocellular carcinoma also supports this view [29]. The findings from our study appear to be consistent with existing literature suggesting that EPHX2 may play a tumor-suppressor-like role in LUAD biology.

*EPHX2* encodes soluble epoxide hydrolase (sEH), an important enzyme that metabolizes epoxyeicosatrienoic acids (EETs) [30]. EET metabolism has been associated with biological processes such as inflammation, vascular homeostasis, oxidative stress, and cellular proliferation [30]. Furthermore, it has been demonstrated that changes in arachidonic acid metabolism and lipid signaling may influence the tumor microenvironment [27]. Therefore, the low EPHX2 expression observed in LUAD tissues may reflect an adaptation associated with metabolic reprogramming in tumor cells and altered lipid homeostasis within the tumor microenvironment [31].

However, *EPHX2* expression was found to be higher in tumor tissues in the GEO GSE10072 dataset. This discrepancy may be attributed to factors such as technical differences between RNA-seq and microarray platforms, sample size, batch effects, patient heterogeneity, and tumor subtypes [32,33]. In addition, GSE10072 represents a single microarray cohort with a relatively limited sample size compared with the larger RNA-seq datasets used in GEPIA2 and TNMplot analyses. Therefore, cohort-specific characteristics or technical variability may have contributed to the opposite expression pattern. Accordingly, it is important to validate these findings in independent clinical cohorts and experimental models. Nevertheless, the observation of generally similar expression trends across different databases supports the overall consistency of the findings regarding EPHX2 expression in LUAD.

The finding that high *EPHX2* expression is associated with better overall survival across most survival analyses supports its potential prognostic relevance in LUAD. Consistent results from the Kaplan–Meier Plotter and the Human Protein Atlas data demonstrate that this association can be replicated across different platforms. Additionally, *EPHX2* expression remained statistically significant in the multivariate Cox regression analysis after adjustment for stage, sex, and smoking status, although this finding should be interpreted with caution given the relatively modest level of statistical significance. However, the failure to reach statistical significance in the GEPIA2 analysis may stem from differences in datasets, patient numbers, follow-up durations, and analysis algorithms [32]. Nevertheless, the observation of generally similar survival trends across different platforms supports the notion that EPHX2 may be a potential prognostic biomarker for LUAD.

Notably, high *EPHX2* expression was associated with better overall survival particularly in Stage I, N0, and M0 patients. This suggests that *EPHX2* may be more closely associated with early tumor progression and local spread. It is known that tumor metabolism and microenvironmental reprogramming play a significant role in early tumor progression [34]. The loss of prognostic significance in advanced stages may be related to the dominance of different molecular mechanisms during metastatic progression. However, the findings should be interpreted not as direct evidence of metastasis suppression, but as bioinformatics observations supporting a potential association with metastatic biology [31,35].

High EPHX2 expression was also associated with better overall survival across additional clinical subgroups, including patients with negative surgical margins, both sexes, and both smokers and never-smokers, further supporting its potential prognostic relevance. The significant enrichment of lipid metabolism, arachidonic acid metabolism, and detoxification processes in genes correlated with *EPHX2* suggests that *EPHX2* may be involved in metabolic regulation in LUAD biology. The prominence of eicosanoid metabolism, fatty acid metabolism, cytochrome P450-mediated xenobiotic metabolism, and biological oxidation pathways in GO, KEGG, and Reactome analyses supports EPHX2’s association with lipid biochemistry and oxidative metabolism. Additionally, it has previously been reported that the CYP450–EET–sEH axis plays a role in tumor progression and inflammatory processes [31,36]. Given that EPHX2 is an enzyme involved in the metabolism of arachidonic acid derivatives, the findings are consistent with the gene’s known biological functions.

It is also noteworthy that there is an enrichment of arachidonic acid metabolism and EET-related pathways. These pathways are associated with cancer progression, inflammation, vascular regulation, oxidative stress, and the tumor microenvironment [31,36,37]. In recent years, it has been shown that metabolic reprogramming in cancer affects not only energy production but also signal transduction, immune response, and treatment resistance [31]. Therefore, it can be hypothesized that changes in EPHX2 expression may be associated with the metabolic reprogramming of tumor cells. Furthermore, the positive correlations observed with the PPARA/RXR axis also support the possibility that EPHX2 is linked to transcriptional networks associated with metabolic regulation [13].

The prominence of pathways associated with the cell cycle, DNA replication, and DNA repair among those showing negative enrichment suggests that low EPHX2 expression may be associated with a more proliferative and aggressive tumor biology. These findings appear biologically consistent with the observation that high EPHX2 expression is associated with better overall survival.

Correlation analyses further supported the functional enrichment findings, suggesting that EPHX2 may be associated with gene expression patterns related to angiogenesis and tumor progression.

The negative correlation between EPHX2 expression and angiogenesis-related genes such as *VEGFA*, *FLT1*, and *ANGPT2* in correlation analyses suggests that *EPHX2* may be associated with molecular pathways involved in tumor vascularization and angiogenic processes. It is known that the VEGF signaling pathway plays a central role in tumor growth, vascularization, and metastatic progression [35,38]. In particular, it has previously been demonstrated that ANGPT2 is associated with an aggressive tumor phenotype and poor prognosis [39]. Therefore, the association between high EPHX2 expression and lower expression of angiogenesis-related genes may partially explain the better overall survival outcomes observed in our study.

Although several correlations reached statistical significance, their relatively modest correlation coefficients indicate limited effect sizes. Accordingly, statistical significance should be interpreted alongside effect size when assessing the biological relevance of these findings. However, the relatively low correlation coefficients suggest that these associations may reflect indirect or weak biological interactions, potentially mediated through broader metabolic and signaling networks. Therefore, the current findings should be interpreted as potential molecular associations rather than causal mechanisms.

These findings are consistent with the literature on EET metabolism. It has been reported that EETs can enhance angiogenesis by activating the VEGF signaling pathway [40,41,42]. Therefore, it is plausible that the decrease in EPHX2 expression may be associated with angiogenic processes, potentially through alterations in EET metabolism.

The negative correlations observed with *MMP2*, *MMP9*, *TIMP1*, *TIMP2*, and *ITGB1* suggest that *EPHX2* may be linked to biological processes associated with extracellular matrix remodeling, matrix proteolysis, cell adhesion/migration, and invasive tumor behavior. The MMP family and *TIMP1/TIMP2* play important roles in extracellular matrix remodeling, tumor invasion, and metastatic spread [43]. *ITGB1*, on the other hand, plays a significant role in cell adhesion and migration [44]. The inverse relationship between the expression of these genes and *EPHX2* suggests that *EPHX2* may be associated with reduced expression of genes involved in invasion and migration. The absence of a significant association with EMT markers such as *VIM* and *CDH1* suggests that this effect may not occur through the classical EMT program. Therefore, it is plausible that EPHX2’s association with invasive processes may be related to ECM remodeling and cell–microenvironment interactions.

The negative correlation observed with *HIF1A* is also noteworthy. It is known that HIF-1α is one of the key transcription factors regulating angiogenesis by increasing VEGF-A expression under hypoxic conditions [35]. Therefore, it can be hypothesized that low EPHX2 expression may be associated with signaling pathways related to the hypoxic response and angiogenesis.

In addition to downstream mechanisms, correlation analyses of upstream regulatory networks revealed that *EPHX2* expression was significantly associated with genes involved in lipid metabolism, the oxidative stress response, and transcriptional regulation. Notably, EPHX2 expression showed a positive correlation with genes involved in lipid metabolism and nuclear receptor signaling, including *PPARA*, *RXRA*, *RXRB*, and *HNF4A*. Given that EPHX2 is an enzyme involved in arachidonic acid metabolism, these findings support the possibility that the gene may be linked to biological processes related to lipid metabolism [31]. Furthermore, the positive correlation with *NFE2L2* suggests that EPHX2 may be linked to mechanisms of the oxidative stress response and cellular redox balance [45]. The positive correlations identified with *CEBPA* and *STAT3*, on the other hand, suggest that EPHX2 may be associated with transcriptional networks involved in metabolic regulation and inflammation [46]. However, further experimental studies are needed to elucidate the biological significance of these relationships.

STRING-based PPI analyses have revealed network structures that support the findings obtained from correlation analyses. Within the scope of downstream analysis, distinct interaction networks were observed among VEGFA, FLT1, ANGPT2, MMP2, MMP9, TIMP1, TIMP2, ITGB1, TEK, and HIF1A. In particular, the prominence of the VEGFA–FLT1 axis and ANGPT2/TEK interactions supports the possibility that EPHX2 may be linked to angiogenesis-related processes [38]. Additionally, the connections between MMP2/MMP9 and TIMP1/TIMP2 appear consistent with findings regarding EPHX2-associated ECM remodeling and invasive processes [43].

It is noteworthy that strong interactions were observed between *PPARA*, *RXRA*, and *RXRB* in upstream analyses. These genes are known to play central roles in lipid metabolism and nuclear receptor signaling [31]. Furthermore, the fact that transcriptional regulators such as *STAT3*, *NFE2L2*, and *CEBPA* also exhibit strong interactions within the same network suggests that these factors may be associated with EPHX2 expression [45,46]. In particular, the NRF2 pathway is known to be associated with oxidative stress and metabolic adaptation in LUAD [45]. In contrast, EPHX2’s more isolated position within the network suggests that its observed associations may be related to indirect metabolic networks or transcriptional regulatory mechanisms rather than direct protein interactions. This finding is also consistent with the metabolic role of EPHX2, suggesting that its biological effects may be mediated through metabolic pathways and broader regulatory networks rather than extensive direct protein–protein interactions. The identification of arachidonic acid metabolism as the most significantly enriched pathway in the STRING-based KEGG enrichment analysis further supports these findings [37].

The hypothesis-generating model derived from the integrated evaluation of correlation, STRING/PPI, and functional enrichment analyses suggests that EPHX2 may be associated with upstream transcriptional regulators associated with lipid metabolism, oxidative stress, and inflammatory signaling pathways; furthermore, potentially through downstream effectors, EPHX2 may also be associated with angiogenesis, extracellular matrix remodeling, hypoxia-related signaling, and metastatic progression processes. These proposed upstream and downstream relationships are based on correlation and network analyses and should not be interpreted as evidence of direct regulatory relationships. Further experimental studies are needed to validate these hypotheses. In this context, EPHX2 may represent a potential prognostic biomarker and contribute to our understanding of metabolic reprogramming in LUAD.

In the present study, the association between high EPHX2 expression and better overall survival suggests that EPHX2-related metabolic homeostasis may be biologically relevant in LUAD progression. Since *EPHX2* encodes soluble epoxide hydrolase (sEH), which is involved in arachidonic acid metabolism, pharmacological targeting of EPHX2-associated metabolic networks may have translational relevance in LUAD [30,36,37]. In particular, the growing recognition of pathways related to metabolic reprogramming, inflammatory signaling, and the tumor microenvironment as potential therapeutic targets suggests that EPHX2-associated molecular pathways may also represent promising areas for future investigation [36,40,42]. Nevertheless, further experimental studies and prospective clinical validations are required to clarify the potential clinical implications of these findings.

This study has several limitations. First, the study is based on retrospective bioinformatics analyses, and the findings were evaluated using transcriptomic and proteomic data obtained from publicly available databases. Therefore, the observed associations do not demonstrate causal mechanisms and should be interpreted at a correlational level. In addition, functional in vitro/in vivo experiments directly evaluating the biological functions of EPHX2, as well as prospective clinical validation, were not performed in this study. In addition, because the present study relied on publicly available web-based transcriptomic platforms, tumor purity and cell-type composition were not specifically evaluated. Therefore, the reduced EPHX2 expression observed in tumor tissues may partly reflect differences in tumor purity and cell-type composition, and this possibility should be considered when interpreting the findings. Furthermore, the relatively low correlation coefficients suggest that the observed associations may occur through indirect or complex regulatory networks. Therefore, further validation of these findings in larger independent cohorts and experimental studies is needed.

## 5. Conclusions

In conclusion, this study suggests that EPHX2 expression may have prognostic significance in LUAD and that high EPHX2 expression is associated with better overall survival. Functional enrichment, correlation, and STRING/PPI analyses suggest that EPHX2 may be associated with biological processes particularly related to lipid metabolism, arachidonic acid metabolism, oxidative stress response, angiogenesis, and extracellular matrix remodeling. The findings also suggest that EPHX2 may be associated with regulatory pathways related to metabolic regulation and tumor progression.

In this context, EPHX2 may represent a potential prognostic biomarker and contribute to our understanding of metabolic reprogramming in LUAD. Nevertheless, further experimental studies and independent clinical validations are needed to clarify the biological and clinical significance of these associations. Overall, the integration of transcriptomic, proteomic, survival, and pathway-level findings provides a broader framework for understanding the potential metabolic relevance of EPHX2 in LUAD biology.

## Figures and Tables

**Table 1:** Univariate Cox regression analysis of EPHX2 according to clinicopathological subgroups in LUAD.

Variable	Subgroup	*n*	HR (95% CI)	*p*-Value
**Overall**	All patients	1161	0.62 (0.52–0.74)	<0.001
**Stage**	Stage I	370	0.42 (0.28–0.63)	<0.001
	Stage II	136	0.78 (0.48–1.27)	0.32
	Stage III	24	1.03 (0.37–2.89)	0.95
	Stage IV	4	Not estimable	—
**Grade**	Grade 1	166	0.91 (0.61–1.36)	0.65
	Grade 2	209	0.77 (0.53–1.12)	0.17
	Grade 3	60	1.06 (0.49–2.26)	0.89
**T stage**	T1	272	0.72 (0.49–1.05)	0.083
	T2	357	0.79 (0.60–1.05)	0.11
	T3	32	0.78 (0.36–1.70)	0.53
	T4	11	Not estimable	—
**N stage**	N0	485	0.72 (0.54–0.95)	0.018
	N1	130	0.60 (0.39–0.92)	0.019
	N2	56	0.96 (0.55–1.68)	0.88
**M stage**	M0	232	0.54 (0.36–0.80)	0.002
	M1	10	Not estimable	—
**Gender**	Female	537	0.58 (0.44–0.77)	<0.001
	Male	566	0.67 (0.53–0.84)	<0.001
**Smoking status**	Never-smoker	192	0.51 (0.27–0.93)	0.026
	Smoker	546	0.69 (0.53–0.89)	0.005
**Surgical margin**	Negative (R0)	636	0.59 (0.46–0.75)	<0.001
**Chemotherapy**	Yes	125	0.82 (0.52–1.30)	0.40
	No	361	0.89 (0.66–1.20)	0.45
**Radiotherapy**	No	363	0.89 (0.66–1.20)	0.46
	Yes	65	0.59 (0.34–1.05)	0.069

Note: *n* values indicate the number of samples included in each subgroup analysis. Analyses could not be performed for the Stage IV, T4, and M1 subgroups due to insufficient sample size. *p*-values were based on the log-rank test.

## Data Availability

The datasets analyzed during this study are publicly available in the TCGA, GEO, CPTAC, GEPIA2, UALCAN, LinkedOmics, STRING, and Kaplan–Meier Plotter databases.

## Supplementary Materials

The following supporting information can be downloaded at: https://www.mdpi.com/article/10.3390/genes17080961/s1. Figure S1. TNMplot microarray analysis of EPHX2 expression in normal, tumor, and metastatic lung tissues. Figure S2. Human Protein Atlas protein expression analysis of EPHX2 in LUAD. Figure S3. STRING-based downstream protein–protein interaction (PPI) network associated with EPHX2 in LUAD. Table S1. Summary of the datasets, analyses, and analysis parameters used in this study. Table S2. Genes showing statistically significant correlations with EPHX2 expression in LUAD.

## Abbreviations

ANGPT2	Angiopoietin-2
CPTAC	Clinical Proteomic Tumor Analysis Consortium
DHETs	Dihydroxyeicosatrienoic acids
ECM	Extracellular matrix
EETs:	Epoxyeicosatrienoic acids
EMT:	Epithelial–mesenchymal transition
EPHX2	Epoxide hydrolase 2
FDR	False discovery rate
FLT1	Fms-related receptor tyrosine kinase 1
GEO	Gene Expression Omnibus
GEPIA2	Gene Expression Profiling Interactive Analysis 2
GO	Gene Ontology
GSEA	Gene set enrichment analysis
GTEx	Genotype-Tissue Expression
HIF1A	Hypoxia-inducible factor 1-alpha
HR	Hazard ratio
KEGG	Kyoto Encyclopedia of Genes and Genomes
LUAD	Lung adenocarcinoma
MMP	Matrix metalloproteinase
mTOR	Mammalian target of rapamycin
NES	Normalized enrichment score
NFE2L2	Nuclear factor erythroid 2-related factor 2
NSCLC	Non-small-cell lung cancer
OS	Overall survival
PPARA	Peroxisome proliferator-activated receptor alpha
PPARG	Peroxisome proliferator-activated receptor gamma
PPI	Protein–protein interaction
RXRA	Retinoid X receptor alpha
RXRB	Retinoid X receptor beta
sEH	Soluble epoxide hydrolase
STAT3	Signal transducer and activator of transcription 3
STRING	Search Tool for the Retrieval of Interacting Genes/Proteins
TCGA	The Cancer Genome Atlas
TEK	TEK receptor tyrosine kinase
TIMP	Tissue inhibitor of metalloproteinase
TNM	Tumor–Node–Metastasis
TPM	Transcripts per million
UALCAN	University of Alabama at Birmingham Cancer data analysis portal
VEGFA	Vascular endothelial growth factor A
