# Supplementary material for: EPHX2 Expression and Its Association with Prognosis, Metabolic Regulation, and Metastasis-Related Pathways in Lung Adenocarcinoma
Source: Genes (Basel). 2026 Aug 16;17(8):961. doi: 10.3390/genes17080961 (PMC13512667; doi:10.3390/genes17080961)
Supplement: Supplementary file 1 [file genes-17-00961-s001.zip › Table S1.pdf]

Supplementary Table S1. Summary of the datasets, analyses, and analysis parameters used in this study.

Platform/Database	Dataset	Analysis	Parameters/Settings
TNMplot	LUAD RNA-seq	Gene expression	Matched normal vs. tumor tissues
TNMplot	Lung microarray dataset	Gene expression	Normal, tumor, and metastatic tissues
GEPIA2	TCGA-LUAD + GTEx	Gene expression	$\|log2FC\| = 1$; *p*-value cutoff = 0.01
GEO (GSE10072)	GSE10072	Differential expression	GEO2R; adjusted $p < 0.05$
Human Protein Atlas	LUAD	Protein expression	Default platform settings
UALCAN/CPTAC	CPTAC LUAD	Protein expression	Default platform settings
Kaplan–Meier Plotter	LUAD	Overall survival	Median cutoff; JetSet best probe; probe ID: 209368_at
GEPIA2	LUAD	Overall survival	Median cutoff (50%/50%)
Human Protein Atlas	LUAD	Overall survival	Default platform settings
LinkedOmics/WebGestalt	TCGA-LUAD	Co-expression analysis and GSEA	Pearson correlation; GO, KEGG, Reactome; FDR correction
GEPIA2	LUAD	Correlation analysis	Spearman correlation
STRING v12.0	Candidate genes	PPI network and KEGG pathway enrichment	Interaction score = 0.700; experimental, database, and co-expression evidence; no network expansion
