# Supplementary material for: EPHX2 Expression and Its Association with Prognosis, Metabolic Regulation, and Metastasis-Related Pathways in Lung Adenocarcinoma
Source: Genes (Basel). 2026 Aug 16;17(8):961. doi: 10.3390/genes17080961 (PMC13512667; doi:10.3390/genes17080961)
Supplement: Supplementary file 1 [file genes-17-00961-s001.zip › Table S2.pdf]

Supplementary Table S2. Genes showing statistically significant correlations with EPHX2 expression in LUAD.

Gene	Spearman's *r*	p-value
**VEGFA**	-0.11	0.012
**FLT1**	-0.11	0.019
**ANGPT2**	-0.19	<0.001
**MMP2**	-0.095	0.037
**MMP9**	-0.20	<0.001
**HIF1A**	-0.16	<0.001
**RAF1**	0.23	<0.001
**MAPK3**	0.15	<0.001
**TEK**	0.24	<0.001
**PTK2**	0.11	0.019
**ITGB1**	-0.26	<0.001
**TIMP1**	-0.22	<0.001
**TIMP2**	-0.10	0.025
**CEBPA**	0.35	<0.001
**STAT3**	0.29	<0.001
**RXRB**	0.27	<0.001
**RXRA**	0.26	<0.001
**NFE2L2**	0.25	<0.001
**PPARA**	0.24	<0.001
**SP1**	0.14	0.003
**HNF4A**	0.14	0.003
